# Knowing What You Know in Brain Segmentation Using Bayesian Deep Neural Networks

**DOI:** 10.3389/fninf.2019.00067

**Published:** 2019-10-17

**Authors:** Patrick McClure, Nao Rho, John A. Lee, Jakub R. Kaczmarzyk, Charles Y. Zheng, Satrajit S. Ghosh, Dylan M. Nielson, Adam G. Thomas, Peter Bandettini, Francisco Pereira

**Affiliations:** ^1^Machine Learning Team, National Institute of Mental Health, Bethesda, MD, United States; ^2^Section on Functional Imaging Methods, National Institute of Mental Health, Bethesda, MD, United States; ^3^Data Sharing and Science Team, National Institute of Mental Health, Bethesda, MD, United States; ^4^McGovern Institute for Brain Research, Massachusetts Institute of Technology, Cambridge, MA, United States

**Keywords:** brain segmentation, deep learning, magnetic resonance imaging, Bayesian neural networks, variational inference, automated quality control

## Abstract

In this paper, we describe a Bayesian deep neural network (DNN) for predicting FreeSurfer segmentations of structural MRI volumes, in minutes rather than hours. The network was trained and evaluated on a large dataset (*n* = 11,480), obtained by combining data from more than a hundred different sites, and also evaluated on another completely held-out dataset (*n* = 418). The network was trained using a novel spike-and-slab dropout-based variational inference approach. We show that, on these datasets, the proposed Bayesian DNN outperforms previously proposed methods, in terms of the similarity between the segmentation predictions and the FreeSurfer labels, and the usefulness of the estimate uncertainty of these predictions. In particular, we demonstrated that the prediction uncertainty of this network at each voxel is a good indicator of whether the network has made an error and that the uncertainty across the whole brain can predict the manual quality control ratings of a scan. The proposed Bayesian DNN method should be applicable to any new network architecture for addressing the segmentation problem.

## 1. Introduction

Identifying which voxels in a structural magnetic resonance imaging (sMRI) volume correspond -to different brain structures (i.e., segmentation) is an essential processing step in neuroimaging analyses. These segmentations are often generated using the FreeSurfer package (Fischl, 2012), a process which can take a day or more for each subject (FreeSurfer, 2018). The computational resources for doing this at a scale of hundreds to thousands of subjects are beyond the capabilities of the computational resources available to most researchers. This has led to an interest in the use of deep neural networks as a general approach for learning to predict the outcome of a processing task, given the input data, in a much shorter time period than the processing would normally take. In particular, several deep neural networks have been trained to perform segmentation of brain sMRI volumes (Ronneberger et al., 2015; Fedorov et al., 2017a,b; Li et al., 2017; Dolz et al., 2018; Roy et al., 2018b), taking between a few seconds and a few minutes per volume. These networks predict a manual or an automated segmentation from the structural volumes [Fedorov et al. (2017a,b), Dolz et al. (2018), and Roy et al. (2018b) used FreeSurfer, Petersen et al. (2010) used GIF (Cardoso et al., 2015), and Rajchl et al. (2018) used FSL (Jenkinson et al., 2012), SPM (Friston et al., 1994) and MALP-EM (Ledig et al., 2015)].

These networks, however, have been trained on a limited number (on the order of hundreds or low thousands) of examples from a limited number of sites (i.e., locations and/or scanners), which can lead to poor cross-site generalization for complex segmentation tasks with a large number of classes (McClure et al., 2018). This includes several of the recent DNNs proposed for fine-grain sMRI segmentation. (Note: We focus on DNNs which predict >30 classes.)

Roy et al. (2018b) performed 33 class segmentation using 581 sMRI volumes from the IXI dataset to train an initial model and then fine-tuned on 28 volumes from the MALC dataset (Marcus et al., 2007). They showed an ~9.4% average Dice loss on out-of-site data from the ADNI-29 (Mueller et al., 2005), CANDI (Kennedy et al., 2012), and IBSR (Rohlfing, 2012) datasets. Fedorov et al. (2017a) used 770 sMRI volumes from HCP (Van Essen et al., 2013) to train an initial model and then fine-tuned on 7 volumes from the FBIRN dataset (Keator et al., 2016). Li et al. (2017) performed a 160 class segmentation using 443 sMRI volumes from the ADNI dataset (Petersen et al., 2010) for training. Rajchl et al. (2018) used 5,000 sMRI volumes from the UK Biobank Sudlow et al. (2015) dataset to train a model by using multi-task learning to simultaneously predict 4 SPM labels, 17 FSL labels, and 139 MALP-EM labels. However, Fedorov et al. (2017a), Li et al. (2017), and Rajchl et al. (2018) did not report test results for sites that where not used during training.

These results show that it is possible to train a neural network to carry out segmentation of a sMRI volume. However, they provide a limited indication of whether such a network would work on data from any new site not encountered in training. While fine-tuning on labeled data from new sites can improve performance, even while using small amounts of data (Fedorov et al., 2017a; McClure et al., 2018; Roy et al., 2018b), a robust neural network segmentation tool should generalize to new sites without any further effort.

As part of the process of adding segmentation capabilities to the “Nobrainer” tool^1^, we trained a network to predict FreeSurfer segmentations given a training set of ~10,000 sMRI volumes. This paper describes this process, as well as a quantitative and qualitative evaluation of the performance of the resulting model.

Beyond the segmentation performance of the network, a second aspect of interest to us is to understand whether it is feasible for a network to indicate how confident it is about its prediction at each location in the brain. We expect the network to make errors, be it because of noise, unusual positioning of the brain, very different contrast than what it was trained on, etc. Because our model is probabilistic and seeks to learn uncertainties, we expect it to be less confident in its predictions in such cases. It is also possible that, for certain locations, there are very similar brain structures labeled as different regions in different people. In such locations, there would be a limit to how well the network could perform, the Bayes error rate (Hastie et al., 2005). Additionally, the network should be less confident for examples that are very different from those seen in the training set (e.g., contain large artifacts). While prediction uncertainty can be computed for standard neural networks, as done by Dolz et al. (2018), these uncertainty estimates are often overconfident (Guo et al., 2017; McClure and Kriegeskorte, 2017). Bayesian neural networks (BNNs) have been proposed as a solution to this issue. One popular BNN approach is Monte-Carlo (MC) Bernoulli Dropout (Srivastava et al., 2014; Gal and Ghahramani, 2016). Using this method, Li et al. (2017) and Roy et al. (2018a) showed that the segmentation performance of the BNN predictions was better for voxels with low dropout sampling-based uncertainties and that injected input noise can lead to increased uncertainty. Roy et al. (2018a) also found that using MC Bernoulli dropout decreased the drop in segmentation performance from 9.4% to 7.8% on average when testing on data from new sites compared to Roy et al. (2018b). However, MC Bernoulli dropout does not learn dropout probabilities from data, which can lead to not properly modeling the uncertainty of the predicted segmentation. Recent works has shown that these dropout probabilities can be learned using a concrete relaxation (Gal et al., 2017). Additionally, learning individual uncertainties for each weight has been shown to be beneficial for many purposes (e.g., pruning and continual learning) (Blundell et al., 2015; McClure et al., 2018; Nguyen et al., 2018). In this paper, we propose using both learned dropout uncertainties and individual weight uncertainties.

Finally, we test the hypothesis that overall prediction uncertainty across an entire image reflects its “quality,” as measured by human quality control (QC) scores. Given the effort required to produce such scores, there have been multiple attempts to either crowdsource the process (Keshavan et al., 2018) or automate it (Esteban et al., 2017). The latter, in particular, does not rely on segmentation information, so we believe it is worthwhile to test whether uncertainty derived from segmentation is more effective.

## 2. Methods

### 2.1. Data

#### 2.1.1. Imaging Datasets

We combined several datasets (Table 1), many which themselves contain data from multiple sites, into a single dataset with 11,480 T1 sMRI volumes. In-site validation and test sets were created from the combined dataset using a 80-10-10 training-validation-test split. This resulted in a training set of 9,184 volumes, a validation set of 1,148 volumes, and a test set of 1,148 volumes. The training set was used for training the networks, the validation set for setting DNN hyperparameters (e.g., Bernoulli dropout probabilities), and the test set was used for evaluating the performance of the DNNs on new data from the same sites that were used for training.

**Table 1:** The number of examples used from different datasets.

**Dataset**	**Number of examples**
CoRR (Zuo et al., 2014)	3,039
OpenfMRI (Poldrack et al., 2013)	1,873
NKI (Nooner et al., 2012)	1,136
SLIM (Liu et al., 2017)	1,003
ABIDE (Di Martino et al., 2014)	992
HCP (Van Essen et al., 2013)	956
ADHD200 (Bellec et al., 2017)	719
CMI (Alexander et al., 2017)	611
SALD (Wei et al., 2018)	477
Buckner (Biswal et al., 2010)	183
HBNSSI (O'Connor et al., 2017)	178
GSP (Holmes et al., 2015)	152
Haxby (Haxby et al., 2011; Nastase et al., 2017)	55
Gobbini (di Oleggio Castello et al., 2017)	51
ICBM (Mazziotta et al., 2001)	45
Barrios (Vzquez et al., 2016)	10

We additionally used 418 sMRI volumes from the NNDSP dataset (Lee et al., 2018) as a held-out dataset to test generalization of the network to an unseen site. In addition to sMRI volumes, each NNDSP sMRI volume was given a QC score from 1 to 4, higher scores corresponding to worse scan quality, by two raters (3 if values differed by more than 1), as described in Blumenthal et al. (2002). If a volume had a QC score greater than 2, it was labeled as a bad quality scan; otherwise, the scan was labeled as a good quality scan.

#### 2.1.2. Segmentation Target

We computed 50-class FreeSurfer (Fischl, 2012) segmentations, as in Fedorov et al. (2017a), for all subjects in each of the datasets described earlier. These were used as the labels for prediction. Although, FreeSurfer segmentations may not be perfectly correct, as compared to manual, expert segmentations, using them allowed us to create a large training dataset, as one could not feasibly label it by hand. FreeSurfer trained networks can also outperform FreeSurfer segmentations when compared to expert segmentations (Roy et al., 2018b). The trained network could be fine-tuned with expert small amounts of labeled data, which would likely improve the results (McClure et al., 2018; Roy et al., 2018b).

#### 2.1.3. Data Pre-processing

The sMRI volumes were resampled to 1mm isotropic cubic volumes of 256 voxels per side and the voxel intensities were normalized according to Freesurfer's mri_convert with the conform flag. After resampling, input volumes were individually z-scored across voxels. We then split each sMRI volume into 512 non-overlapping 32 × 32 × 32 sub-volumes, similarly to Fedorov et al. (2017b) and Fedorov et al. (2017a), to be used as inputs for the neural network. The prediction target is the corresponding segmentation sub-volume. This resulted in 512 pairs (**x**, **y**), of sMRI and label sub-volumes, respectively, for each sMRI volume.

### 2.2. Convolutional Neural Network

#### 2.2.1. Architecture

Several deep neural network architectures have been proposed for brain segmentation, such as U-net (Ronneberger et al., 2015), QuickNAT (Roy et al., 2018b), HighResNet (Li et al., 2017), and MeshNet (Fedorov et al., 2017a,b). We chose MeshNet because of its relatively simple structure, its lower number of learned parameters, and its competitive performance, since the computational cost of Bayesian neural networks scales based on structural complexity and number of parameters.

MeshNet uses dilated convolutional layers (Yu and Koltun, 2015) due to the 3D structural nature of sMRI data. Applying a discrete volumetric dilated convolutional layer to one input channel for one weight filter can be expressed as:

(1) $$\begin{array}{l} {\left(W_{f}{*}_{l}h\right)}_{i,j,k}\;=\;\sum_{\tilde{i}=-a}^{a}\sum_{\tilde{j}=-b}^{b}\sum_{\tilde{k}=-c}^{c}w_{f,\tilde{i},\tilde{j},\tilde{k}}h_{i-l\tilde{i},j-l\tilde{j},k-l\tilde{k}}={\left(W_{f}{*}_{l}h\right)}_{v}\\ \;=\sum_{t\in W_{abc}}w_{f,t}h_{v-lt}. \end{array}$$

where *h* is the input to the layer, *a*, *b*, and *c* are the bounds for the *i*, *j*, and *k* axes of the filter with weights **w**_*f*_ (*i, j, k*) is the voxel, **v**, where the convolution is computed. The set of indices for the elements of **w**_*f*_ can be defined as $W_{abc}=\left\{-a,\ldots ,a\right\}\times \left\{-b,\ldots ,b\right\}\times \left\{-c,\ldots ,c\right\}$. The dilation factor, number of filters, and other details of the MeshNet-like architecture that we used for all experiments is shown in Table 2. Note that we increased the number of filters per layer from 72 to 96, compared to Fedorov et al. (2017a) and McClure et al. (2018), since we greatly increased the number of training volumes.

**Table 2:** The MeshNet dilated convolutional neural network architecture used for brain segmentation.

**Layer**	**Filter**	**Padding**	**Dilation (*l*)**	**Non-linearity**
1	96 × 3^3^	1	1	ReLU
2	96 × 3^3^	1	1	ReLU
3	96 × 3^3^	1	1	ReLU
4	96 × 3^3^	2	2	ReLU
5	96 × 3^3^	4	4	ReLU
6	96 × 3^3^	8	8	ReLU
7	96 × 3^3^	1	1	ReLU
8	50 × 1^3^	0	1	Softmax

#### 2.2.2. Maximum a Posteriori Estimation

When training a neural network, the weights of the network, **w**, are often learned using maximum likelihood estimation (MLE). For MLE, logp(D|w) is maximized where $D=\left\{\left({\text{x}}_{1},{\text{y}}_{1}\right),\ldots ,\left({\text{x}}_{N},{\text{y}}_{N}\right)\right\}$ is the training dataset and (**x**_*n*_, **y**_*n*_) is the *n*th input-output example. This often overfits, however, so we used a prior on the network weights, *p*(**w**), to obtain a maximum a posteriori (MAP) estimate, by optimizing logp(w|D):

(2) $$\begin{array}{r} {\text{w}}^{*}=\underset{\text{w}}{\text{argmax}}\overset{N}{\underset{n=1}{\sum }}\log p\left({\text{y}}_{n}|{\text{x}}_{n},\text{w}\right)+\log p\left(\text{w}\right). \end{array}$$

We used a fully factorized Gaussian prior (i.e., $p(w_{f,\tilde{i},\tilde{j},\tilde{k}})=N(0,1))$. This results in the MAP weights being learned by minimizing the softmax cross-entropy with L2 regularization. At test time, this point estimate approximation, w^*^, is used to make a prediction for new examples:

(3) $$\begin{array}{r} p\left({\text{y}}_{test}|{\text{x}}_{test}\right)\approx p\left({\text{y}}_{test}|{\text{x}}_{test},{\text{w}}^{*}\right) \end{array}$$

#### 2.2.3. Approximate Bayesian Inference

In Bayesian inference for neural networks, a distribution of possible weights is learned instead of just a MAP point estimate. Using Bayes' rule, p(w|D)=p(D|w)p(w)/p(D), where *p*(**w**) is the prior over weights. However, directly computing the posterior, p(w|D), is often intractable, particularly for DNNs. As a result, an approximate inference method must be used.

One of the most popular approximate inference methods for neural networks is variational inference, since it scales well to large DNNs. In variational inference, the posterior distribution p(w|D) is approximated by a learned variational distribution of weights *q*_θ_(**w**), with learnable parameters θ. This approximation is enforced by minimizing the Kullback-Leibler divergence (KL) between *q*_θ_(**w**), and the true posterior, p(w|D), $\text{KL}\left[q_{\theta }\left(\text{w}\right)||p\left(\text{w}|D\right)\right]$, which measures how *q*_θ_(**w**) differs from p(w|D) using relative entropy. This is equivalent to maximizing the variational lower bound (Hinton and Van Camp, 1993; Graves, 2011; Blundell et al., 2015; Kingma et al., 2015; Gal and Ghahramani, 2016; Louizos and Welling, 2017; Molchanov et al., 2017), also known as the evidence lower bound (ELBO),

(4) $$\begin{array}{r} L_{ELBO}\left(\theta \right)=L_{D}\left(\theta \right)-L_{KL}\left(\theta \right), \end{array}$$

where $L_{D}\left(\theta \right)$ is

(5) $$\begin{array}{r} L_{D}\left(\theta \right)=\overset{N}{\underset{n=1}{\sum }}{𝔼}_{q_{\theta }\left(\text{w}\right)}\left[\log p\left({\text{y}}_{n}|{\text{x}}_{n},\text{w}\right)\right] \end{array}$$

and $L_{KL}\left(\theta \right)$ is the KL divergence between the variational distribution of weights and the prior,

(6) $$\begin{array}{r} L_{KL}\left(\theta \right)=\text{KL}\left[q_{\theta }\left(\text{w}\right)||p\left(\text{w}\right)\right], \end{array}$$

which measures how *q*_θ_(**w**) differs from *p*(**w**) using relative entropy.

Maximizing $L_{D}$ seeks to learn a *q*_θ_(**w**) that explains the training data, while minimizing *L*_*KL*_ (i.e., keeping *q*_θ_(**w**) close to *p*(**w**)) prevents learning a *q*_θ_(**w**) that overfits to the training data.

The objective function in Equation (4) is usually impractical to compute for deep neural networks, due to both: (1) being a full-batch approach and (2) integrating over *q*_θ_(**w**). (1) is often dealt with by using stochastic mini-batch optimization (Robbins and Monro, 1951) and (2) is often approximated using Monte Carlo sampling. As discussed in Graves (2011) and Kingma et al. (2015), these methods can be used to perform stochastic gradient variational Bayes (SGVB) in deep neural networks. For each parameter update, an unbiased estimate of $\nabla_{\theta }L_{D}$ for a mini-batch, {(**x**_1_, **y**_1_), …, (**x**_*M*_, **y**_*M*_)}, is calculated using one weight sample, **w**_*m*_, from *q*_θ_(**w**) for each mini-batch example. This results in the following approximation to Equation (4):

(7) $$\begin{array}{r} L_{ELBO}\left(\theta \right)\approx L_{D}^{SGVB}\left(\theta \right)-L_{KL}\left(\theta \right), \end{array}$$

where

(8) $$\begin{array}{r} L_{D}\left(\theta \right)\approx L_{D}^{SGVB}\left(\theta \right)=\frac{N}{M}\overset{M}{\underset{m=1}{\sum }}\log p\left({\text{y}}_{m}|{\text{x}}_{m},{\text{w}}_{m}\right). \end{array}$$

**Figure 1:**
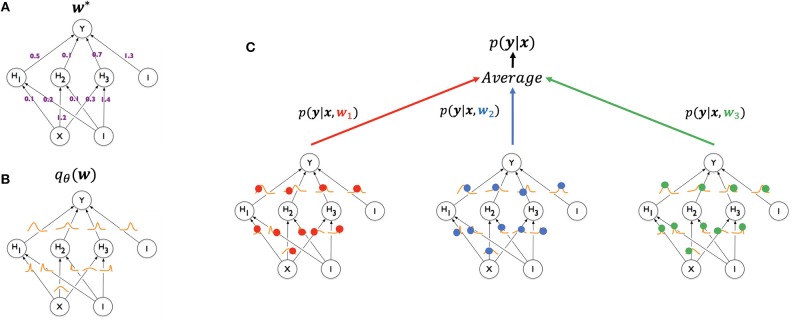
Illustration of generating a prediction from a Bayesian neural network using Monte Carlo sampling (modified from Blundell et al., 2015). A standard neural network (**A**, top left) has one weight for each of its connections (w^*^), learned from the training set and used in generating a prediction for a test example. A Bayesian neural network (**B**, bottom left) has, instead, a posterior distribution for each weight, parameterized by theta (*q*_θ_(**w**)). The process of training starts with an assigned prior distribution for each weight, and returns an approximate posterior distribution. At test time (**C**, right), a weight sample **w**_1_ (red) is drawn from the posterior distribution of the weights, and the resulting network is used to generate a prediction *p*(**y**|**x**, **w**_1_) for an example *x*. The same can be done for samples **w**_2_ (blue) and **w**_3_ (green), yielding predictions *p*(**y**|**x**, **w**_2_) and *p*(**y**|**x**, **w**_3_), respectively. The three networks are treated as an ensemble and their predictions averaged.

At test time, the weights, **w** would ideally be marginalized out, *p*(**y**_*test*_|**x**_*test*_) = ∫*p*(**y**_*test*_|**x**_*test*_, **w**)*q*_θ_(**w**)*d***w**, when making a prediction for a new example. However, this is often impractical to compute for DNNs, so a Monte-Carlo approximation is often used. This results in the prediction of a new example being made by averaging the predictions of multiple weight samples from *q*_θ_(**w**) (Figure 1):

(9) $$\begin{array}{r} p\left({\text{y}}_{test}|{\text{x}}_{test}\right)\approx \frac{1}{N_{MC}}\overset{N_{MC}}{\underset{n}{\sum }}p\left({\text{y}}_{test}|{\text{x}}_{test},{\text{w}}_{n}\right) \end{array}$$

where **w**_*n*_ ~ *q*_θ_(**w**).

##### 2.2.3.1. MC Bernoulli dropout

For MC Bernoulli dropout (BD) (Gal and Ghahramani, 2016), we drew weights from *q*_θ_(**w**) by drawing a Bernoulli random variable (*b*_*i, j, k*_ ~ *Bern*(*p*_*l*_)), where *i, j, k* are the indices of the volume axes, for every element of the layer, *l*, input, **h**, and then elementwise multiplying **b** and **h** before applying the next dilated convolutional layer. This effectively sets the filter weights to zero when applied to a dropped element. Gal and Ghahramani (2016) approximated the KLD between this Bernoulli variational distribution and a zero-mean Gaussian by replacing the variational distribution with a mixture of Gaussians, resulting in an L2-like penalty. However, this can lead to pathological behavior due to Bernoulli distributions not having support over all real numbers (Hron et al., 2018). In Bernoulli dropout, *p*_*l*_ codes for the uncertainty of the weights and is often set layerwise via hyperparameter search (for our experiments, we found the best value of *p* to be 0.9 after searching over the values of 0.95, 0.9, 0.75, and 0.5 using the validation set). However, Bayesian models would ideally learn how uncertain to be for each weight.

##### 2.2.3.2. Spike-and-slab dropout with learned model uncertainty

We propose a form of dropout that both learns the dropout probability for each filter using a concrete relaxation of dropout (Gal et al., 2017), and an individual uncertainty for each weight using fully factorized Gaussian (FFG) filters (Graves, 2011; Blundell et al., 2015; Molchanov et al., 2017; McClure et al., 2018; Nguyen et al., 2018). This is in contrast to previous spike-and-slab dropout methods, which did not learn the model (or epistemic) uncertainty (Der Kiureghian and Ditlevsen, 2009; Kendall and Gal, 2017) from data either by learning the dropout probabilities or by learning the variance parameter of the Gaussian components of the weights (McClure and Kriegeskorte, 2017). In our proposed method, we assume each of the *F* filters are independent (i.e., $p\left(\text{w}\right)=\prod_{f=1}^{F}p\left({\text{w}}_{f}\right)$), as done in previous FFG methods (Graves, 2011; Blundell et al., 2015; Molchanov et al., 2017; McClure et al., 2018; Nguyen et al., 2018). We then decompose each filter into a dropout-based component, *b*_*f*_, and a Gaussian component, **g**_*f*_, such that **w**_*f*_ = *b*_*f*_**g**_*f*_. Per this decomposition, we perform variational inference on the joint distribution of {*b*_1_, …, *b*_*F*_, **g**_1_, …**g**_*F*_}, instead of on p(**w**) directly (Titsias and Lázaro-Gredilla, 2011; McClure and Kriegeskorte, 2017). We then assume each element of **g**_*f*_ is independent (i.e., $p\left({\text{g}}_{f}\right)=\prod_{\text{t}\in W_{abc}}p\left(g_{f,\text{t}}\right)$), and that each weight is Gaussian (i.e., $g_{f,\text{t}}\sim N\left(\mu_{f,\text{t}},\sigma_{f,\text{t}}^{2}\right)$) with learned parameters μ_*f*, **t**_ and σ_*f*, **t**_. Instead of drawing each *b*_*f*_ from *Bern*(*p*_*l*_), we draw them from a concrete distribution (Gal et al., 2017) with a learned dropout probability, *p*_*f*_, for each filter:

(10) $$\begin{array}{r} b_{f}=sigmoid(\frac{1}{t}(\log p_{f}-\log(1-p_{f})+\log u-\log(1-u)) \end{array}$$

where *u* ~ *Unif* (0, 1). This concrete distribution converges to the Bernoulli distribution as the sigmoid scaling parameter, *t*, goes to zero. (In this paper, we used *t* = 0.02). As discussed in Kingma et al. (2015) and Molchanov et al. (2017), randomly sampling each *g*_*f*, **t**_ for each mini-batch example can be computationally expensive, so we used the fact that the sum of independent Gaussian variables is also Gaussian to move the noise from the weights to the convolution operation, as in McClure et al. (2018). For, dilated convolutions and the proposed spike-and-slab variational distribution, this is described by:

(11) $$\begin{array}{r} {\left({\text{w}}_{f}{*}_{l}\text{h}\right)}_{\text{v}}=b_{f}{\left({\text{g}}_{f}{*}_{l}\text{h}\right)}_{\text{v}} \end{array}$$

where

(12) $$\begin{array}{r} {\left({\text{g}}_{f}{*}_{l}\text{h}\right)}_{\text{v}}\sim N\left(\mu_{f,\text{v}}^{*},{\left(\sigma_{f,\text{v}}^{*}\right)}^{2}\right), \end{array}$$

(13) $$\begin{array}{r} \mu_{f,\text{v}}^{*}=\underset{\text{t}\in W_{abc}}{\sum }\mu_{f,\text{t}}h_{\text{v}-l\text{t}}, \end{array}$$

and

(14) $$\begin{array}{r} {\left(\sigma_{f,\text{v}}^{*}\right)}^{2}=\underset{\text{t}\in W_{abc}}{\sum }\sigma_{f,\text{t}}^{2}h_{\text{v}-l\text{t}}^{2}. \end{array}$$

For this spike-and-slab dropout (SSD) implementation, we used a spike-and-slab prior, instead of the Gaussian prior used by Gal and Ghahramani (2016) and Gal et al. (2017). Using a spike-and-slab prior with MC Bernoulli dropout was discussed in Gal (2016), but not implemented. As in the variational distribution, each filter is independent in the prior. Per the spike-and-slab decomposition discussed above, the KL-divergence term of the ELBO can be written as

(15) $$\begin{array}{r} L_{KL}\left(\theta \right)=\overset{F}{\underset{f=1}{\sum }}\text{KL}\left[q_{p_{f}}\left(b_{f}\right)q_{\mu ,\sigma }\left({\text{g}}_{f}\right)||p\left(b_{f}\right)p\left({\text{g}}_{f}\right)\right], \end{array}$$

where $\theta ={⋃}_{f}^{F}{⋃}_{\text{t}\in W_{abc}}\left\{p_{f},\mu_{f,\text{t}},\sigma_{f,\text{t}}\right\}$ are the learned parameters and *p*(*b*_*f*_) and *p*(**g**_*f*_) are priors. Assuming that each weight in a filter is independent, as commonly done in the literature (Graves, 2011; Blundell et al., 2015; Nguyen et al., 2018), allows the term to be rewritten as

(16) $$\begin{array}{r} L_{KL}\left(\theta \right)=\overset{F}{\underset{f=1}{\sum }}\left(\text{KL}\left[q_{p_{f}}\left(b_{f}\right)||p\left(b_{f}\right)\right]+\underset{\text{t}\in W_{abc}}{\sum }\text{KL}\left[q_{\mu ,\sigma }\left(g_{f,\text{t}}\right)||p\left(g_{f,\text{t}}\right)\right]\right). \end{array}$$

For KL[*q*_*p*_*f*__||*p*(*b*_*f*_)], we used the KL-divergence between two Bernoulli distributions,

(17) $$\begin{array}{r} KL\left[q_{p_{f}}\left(b_{f}\right)||p\left(b_{f}\right)\right]=p_{f}\log\frac{p_{f}}{p_{prior}}+\left(1-p_{f}\right)\log\frac{1-p_{f}}{1-p_{prior}}, \end{array}$$

since we used a relatively small sigmoid scaling parameter. Using $p\left(g_{f,\text{t}}\right)=N\left(\mu_{prior},\sigma_{prior}^{2}\right)$,

(18) $$\begin{array}{r} KL\left[q_{\mu ,\sigma }\left(g_{f,\text{t}}\right)||p\left(g_{f,\text{t}}\right)\right]=\log\frac{\sigma_{prior}}{\sigma_{f,\text{t}}}+\frac{\sigma_{f,\text{t}}^{2}+{\left(\mu_{f,\text{t}}-\mu_{prior}\right)}^{2}}{2\sigma_{prior}^{2}}-\frac{1}{2}. \end{array}$$

For this paper, the spike-and-slab prior parameters were set as *p*_*prior*_ = 0.5, μ_*prior*_ = 0, and σ_*prior*_ = 0.1. *p*_*prior*_ = 0.5 corresponds to a maximum entropy prior (i.e., in the absence of new data be maximally uncertain). Alternatively, a *p*_*prior*_ close to 0 is a sparcity prior (i.e., in the absence of data do not use a filter).

### 2.3. Implementation Details

The DNNs were implemented using Tensorflow (Abadi et al., 2016). During training, the parameters of each DNN were updated using Adam (Kingma and Ba, 2015) with an initial learning rate of 1e-4. A mini-batch size of 32 subvolumes was used with data parallelization across 4 12GB NVIDIA Titan X Pascal GPUs was used for training and a mini-batch size of 8 subvolumes on 1 12GB NVIDIA Titan X Pascal GPU was used for validation and testing.

### 2.4. Quantifying Performance

#### 2.4.1. Segmentation Performance Measure

To measure the quality of the produced segmentations, we calculated the Dice coefficient, which is defined by

(19) $$\begin{array}{r} Dice_{c}=\frac{2|{\hat{\text{y}}}_{c}\cdot {\text{y}}_{c}|}{||{\hat{\text{y}}}_{c}|{|}^{2}+||{\text{y}}_{c}|{|}^{2}}=\frac{2TP_{c}}{2TP_{c}+FN_{c}+FP_{c}}, \end{array}$$

where ${\hat{\text{y}}}_{c}$ is the binary segmentation for class *c* produced by a network, **y**_*c*_ is the ground truth produced by FreeSurfer, *TP*_*c*_ is the true positive rate for class *c*, *FN*_*c*_ is the false negative rate for class *c*, and *FP*_*c*_ is the false positive rate for class *c*. We calculate the Dice coefficient separately for each class *c* = 1, …, 50, and average across classes to compute the overall performance of a network for one sMRI volume.

#### 2.4.2. Uncertainty Measure

We quantify the uncertainty of a prediction, *p*(**y**_*m*_|**x**_*m*_), using the aleatoric uncertainty (Der Kiureghian and Ditlevsen, 2009; Kendall and Gal, 2017) for each voxel, **v**. This was measured by the entropy of the softmax across the 50 output classes,

(20) $$\begin{array}{r} H\left(Y_{m,\text{v}}|X_{m}={\text{x}}_{m}\right)=-\overset{50}{\underset{c=1}{\sum }}p\left(y_{m,\text{v}}=c|{\text{x}}_{m}\right)\;\log p\left(y_{m,\text{v}}=c|{\text{x}}_{m}\right), \end{array}$$

We calculated the uncertainty for one sMRI volume by averaging across all output voxels not classified as background (i.e., given the unknown label).

## 3. Results

### 3.1. Segmentation Performance

**Figure 2:**
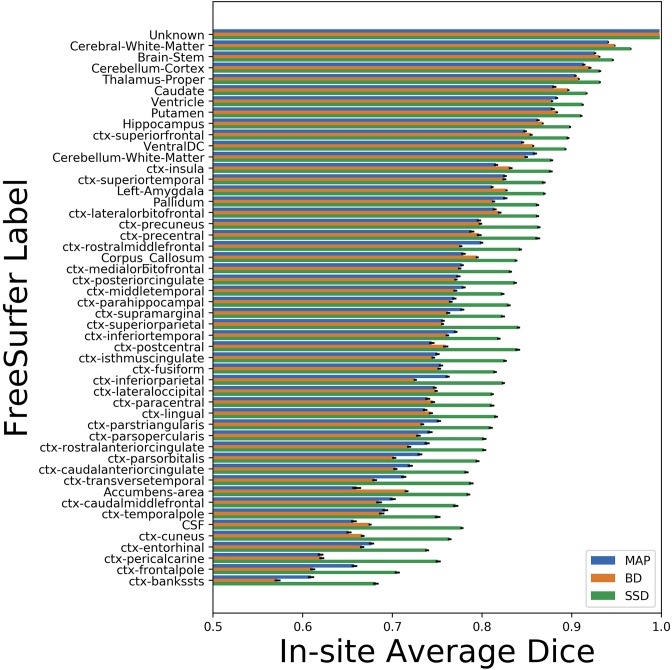
Average Dice scores, sorted in decreasing order by average method performance, and standard errors across in-site test volumes for each label for the maximum a posteriori (MAP), MC Bernoulli dropout (BD), and spike-and-slab dropout (SSD) networks.

**Figure 3:**
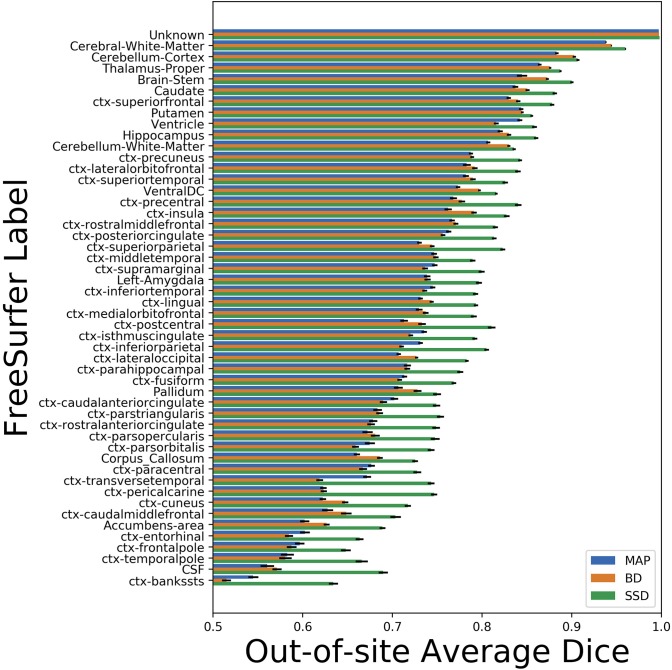
Average Dice scores, sorted in decreasing order by average method performance, and standard errors across out-of-site test volumes for each label for the maximum a posteriori (MAP), MC Bernoulli dropout (BD), and spike-and-slab dropout (SSD) networks.

**Figure 4:**
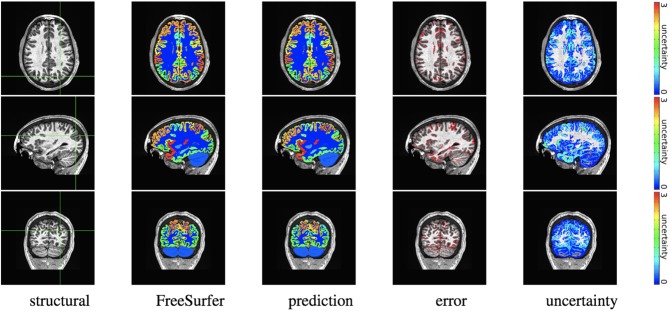
In-site segmentation results for the spike-and-slab dropout (SSD) network for a test subject with average Dice performance. The columns show, respectively, the structural image used as input, the FreeSurfer segmentation used as a prediction target, the prediction made by our network, the voxels where there was a mismatch between prediction and target, and the prediction uncertainty at each voxel.

**Figure 5:**
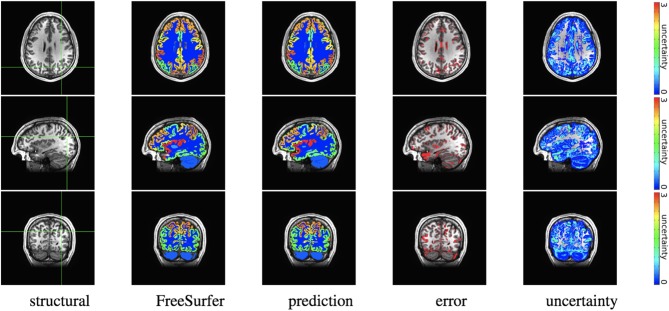
Out-of-site segmentation results for the spike-and-slab dropout (SSD) network for a test subject with average Dice performance. The columns show, respectively, the structural image used as input, the FreeSurfer segmentation used as a prediction target, the prediction made by our network, the voxels where there was a mismatch between prediction and target, and the prediction uncertainty at each voxel.

We trained MAP, MC Bernoulli Dropout (BD), and Spike-and-Slab Dropout (SSD) Meshnet-like CNNs on the 9,184 sMRI volumes in the training set. We then applied our networks to produce segmentations for both the in-site test set and the out-of-site test data. For the BD and SSD networks, 10 MC samples were used for test predictions. The means and standard deviations across volumes for the average Dice across all 50 classes are shown in Table 3. Dice scores for each label for the in-site and out-of-site test sets are shown in Figures 2, 3, respectively. We found that, compared to MAP and BD, SSD significantly increased the Dice for both the in-site (*p* < 1*e* − 6) and out-of-site (*p* < 1*e* − 6) test sets, per a paired *t*-test across test volumes. We found that SSD had a 5.7% drop in performance from the in-site test set to the out-of-site test set, where as the MAP has a drop of 6.2% and BD a drop of 5.4%. This is better than drops of 9.4% and 7.8% on average reported in the literature by Roy et al. (2018b) and Roy et al. (2018a), respectively. In Figures 4, 5, we show selected example segmentations for the SSD network for volumes that have Dice scores similar to the average Dice score across the respective dataset.

**Table 3:** The average and standard deviation of the class Dices across test volumes for the maximum a posteriori (MAP), MC Bernoulli dropout (BD), and spike-and-slab dropout (SSD) network on the in-site and out-of-site test sets.

**Method**	**In-site**	**Out-of-site**
MAP	0.7790 ± 0.0576	0.7333 ± 0.0498
BD	0.7764 ± 0.0506	0.7369 ± 0.0474
SSD	0.8373 ± 0.0471	0.7921 ± 0.0444

### 3.2. Utilizing Uncertainty

#### 3.2.1. Predicting Segmentation Errors From Uncertainty

**Figure 6:**
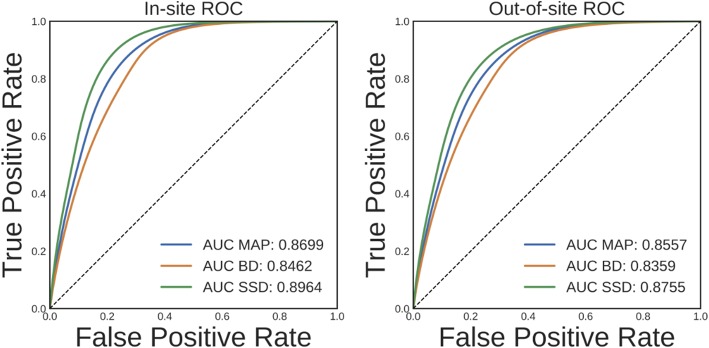
Receiver operating characteristic (ROC) curves for predicting errors for the in-site and out-of-site test sets from the voxel uncertainty of the maximum a posteriori (MAP), MC Bernoulli dropout (BD), and spike-and-slab dropout (SSD) networks.

Ideally, an increase in DNN prediction uncertainty indicates an increase in the likelihood that prediction is incorrect. To evaluate whether this is the case for the trained brain segmentation DNN, we performed a receiver operating characteristic (ROC) analysis. In this analysis, voxels are ranked from most uncertain to least uncertain and one considers, at each rank, what fraction of the voxels were also misclassified by the network. An ROC curve can then be generated by plotting the true positive rate vs. the false negative rate for different uncertainty thresholds used to predict misclassification. The area under this curve (AUC) typically summarizes the results of the ROC analysis. The average ROC and AUCs across volumes for MAP, BD, and SSD for the in-site and out-of-site test sets are shown in Figure 6. Compared to MAP and BD, SSD significantly improved the AUC for both the in-site (*p* < 1*e* − 6) and out-of-site (*p* < 1*e* − 6) test sets, per a paired *t*-test across test set volumes.

#### 3.2.2. Predicting Scan Quality From Uncertainty

**Figure 7:**
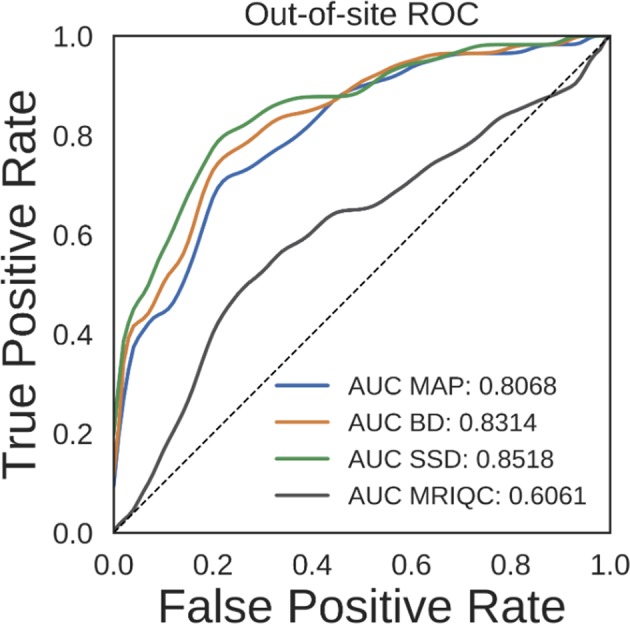
Receiver operating characteristic (ROC) curves for predicting scan quality for the NNDSP out-of-site test set from the average non-background voxel uncertainty of the maximum a posteriori (MAP), MC Bernoulli dropout (BD), and spike-and-slab dropout (SSD) networks and from MRIQC scores.

Ideally, the output uncertainty for inputs not drawn from the training distribution should be relatively high. This could potentially be useful for a variety of applications. One particular application is detection of bad quality sMRI scans, since the segmentation DNN was trained using relatively good quality scans. To test the validity of predicting high vs. low quality scans, we performed an ROC analysis on the held-out NNDSP dataset, where manual quality control ratings are available. We also did the same analysis using MRIQC (v0.10.5) (Esteban et al., 2017), a recently published method that combines a wide range of automated QC algorithms. To statistically test whether any method significantly outperformed the other methods, we performed bootstrap sampling of the AUC for predicting scan quality from average uncertainty by sampling out-of-site test volumes. We performed 10,000 bootstrap samples, each with 418 volumes. The average ROC and AUC for the MAP, BD, SSD, and MRIQC methods are shown in Figure 7. The MAP, BD, and SSD networks all have significantly higher AUCs than MRIQC (*p* = 1.369*e* − 4, *p* = 1.272*e* − 5, and *p* = 1.381*e* − 6, respectively). Additionally, SSD had a significantly higher AUC than both MAP and BD (*p* = 1.156*e* − 3 and *p* = 1.042*e* − 3, respectively).

## 4. Discussion

Segmentation of structures in sMRI volumes is a critical pre-processing step in many neuroimaging analyses. However, these segmentations are currently generated using tools that can take a day or more for each subject (FreeSurfer, 2018), such as FreeSurfer. This computational cost can be prohibitive when scaling analyses up from hundreds to thousands of subjects. DNNs have recently been proposed to perform sMRI segmentation is seconds to minutes. In this paper, we developed a Bayesian DNN, using spike-and-slab dropout, with the goals of increasing the similarity of the DNN's predictions to the FreeSurfer segmentations and generating useful uncertainty estimates for these predictions.

In order to evaluate the proposed Bayesian network, we trained a standard deep neural network (DNN), using MAP estimation, to predict FreeSurfer segmentations from structural MRI (sMRI) volumes. We trained on a little under 10,000 sMRIs, obtained by combining approximately 70 different datasets (many of which, in turn, contain images from several sites, e.g., NKI, ABIDE, ADHD200). We used a separate test set of more than 1,000 sMRIs, drawn from the same datasets. The resulting standard DNN performs at the same level of state-of-the-art networks (Fedorov et al., 2017a). This result, however, was obtained by testing over an order of magnitude more test data, and many more sites, than those papers. We also tested performance on a completely separate dataset (NNDSP) from a site not encountered in training, which contained 418 sMRI volumes. Whereas Dice performance dropped slightly, this was less than what was observed in other studies (Roy et al., 2018a,b); this suggests that we may be achieving better generalization by training on our larger and more diverse dataset, and we plan on testing this on more datasets from novel sites in the future. This is particularly important to us, as this network is meant to be used within an off-the-shelf tool^1^.

We demonstrated that the estimated uncertainty for the prediction at each voxel is a good indicator of whether the standard network makes an error in it, both in-site and out-of-site. The tool that produces the predicted segmentation volume for an input sMRI will also produce an uncertainty volume. We anticipate this being useful at various levels, e.g., to refine other tools that rely on segmentation images, or to improve prediction models based on sMRI data (e.g., modification of calculation of cortical thickness, surface area, voxel selection or weighting in regression (Roy et al., 2018a) or classification models, etc).

We also demonstrated that the average prediction uncertainty across voxels in the brain is an excellent indicator of manual quality control ratings. Furthermore, it outperforms the best existing automated solution (Esteban et al., 2017). Since automation is already used in large repositories (e.g., OpenMRI), we plan on offering our tool as an additional quality control measure.

Finally, we showed that a new Bayesian DNN using spike-and-slab dropout with learned model uncertainty was significantly better than previous approaches. This spike-and-slab method increased segmentation performance and improved the usefulness of output uncertainties compared both to a MAP DNN method and an MC Bernoulli dropout method, which has previously been used in the brain segmentation literature (Li et al., 2017; Roy et al., 2018a). These results show that Bayesian DNNs are a promising method for building brain segmentation and automated sMRI quality control tools. We have also made a version of “Nobrainer,” that incorporates the networks trained and evaluated in this paper, available for download and use within a Singularity/Docker container^2^.

We believe it may be possible to improve this segmentation processing, in that we did not use registration. One option would be to use various techniques for data augmentation (e.g., variation of image contrast, since that is pretty heterogeneous, rotations/translations of existing examples, addition of realistic noise, etc). Another would be to eliminate the need to divide the brain into sub-volumes, which loses some global information; this will become more feasible in GPUs with more memory. Finally, we plan on using post-processing of results (e.g., ensure some coherence between predictions for adjacent voxels, leverage off-the-shelf brain and tissue masking code).

## Data Availability Statement

All datasets generated for this study are included in the manuscript/supplementary files.

## Author Contributions

PM and CZ developed the novel Bayesian neural network method. PM, NR, JK, and SG wrote the code for training and testing the neural networks. PM and NR analyzed the neural network predictions. JL, DN, and AT acquired and preprocessed the magnetic resonance imaging (MRI) data. PM and FP wrote the first manuscript draft. All authors contributed to manuscript revision, read the manuscript, and approved the submitted version.

### Conflict of Interest

The authors declare that the research was conducted in the absence of any commercial or financial relationships that could be construed as a potential conflict of interest.

## Footnotes

^1^https://github.com/neuronets/nobrainer

^2^https://github.com/neuronets/kwyk
